# Enhanced Scar Reduction Using Topical Application of Captopril-containing Silicone Gel in a Rabbit Model of Hypertrophic Scars

**DOI:** 10.7150/ijms.123608

**Published:** 2026-02-26

**Authors:** Jeeyoon Kim, Hyun-mu Jo, Jongweon Shin, Eun Young Rha

**Affiliations:** 1 Department of Plastic and Reconstructive Surgery, College of Medicine, The Catholic University of Korea, Seoul, Republic of Korea.; 2 Research Institute of Medical Science, Eunpyeong St. Mary's Hospital, College of Medicine, The Catholic University of Korea, Seoul, Republic of Korea.

**Keywords:** angiotensin-converting enzyme inhibitors, captopril, cicatrix, medication, silicones

## Abstract

Antihypertensive medications, including angiotensin-converting enzyme (ACE) inhibitors and calcium channel blockers, have been explored for their potential in treating pathological scars. We compared the scar-reducing effects of topical silicone and captopril (an ACE inhibitor) gel used alone and in combination. Eight New Zealand White rabbits with a total of 80 wounds on both ears were used. The animals were assigned to the control, vehicle, silicone, and captopril 50 mg/g and 50 mg/g captopril-containing silicone groups (n = 16 per group). Treatment began on day 28 post-scarring. The scar elevation index (SEI) was measured by histopathology. Scarring was assessed based on fibroblast counts, capillary counts, and epithelial thickness. Ki-67, collagen type I and III, and vascular endothelial growth factor (VEGF) expression was evaluated using immunohistochemistry. SEI, fibroblast and capillary counts, epithelial thickness and collagen type I and III expression were significantly lower in the captopril-containing silicone group than in the other groups (P < 0.001). Ki-67, and VEGF expression also showed significant decreases in the silicone, captopril 50 mg/g, and captopril-containing silicone groups compared with the control and vehicle groups (P < 0.001). Topical application of captopril 50 mg/g alone or in a captopril-containing silicone formulation effectively reduced scar formation, with enhanced effects observed when captopril was combined with silicone. Captopril, therefore, presents a viable alternative to conventional silicone for scar management across various clinical scenarios.

## Introduction

Hypertrophic scars and keloids are characterized by excessive dermal fibrosis and aberrant wound healing resulting from an abnormal healing response to trauma or surgery 1. They are considered dermal fibroproliferative diseases rather than inevitable or unavoidable outcomes 2, as they can affect patients both physically and psychologically, impairing quality of life and posing significant challenges for clinicians. Traditional modalities used to treat and prevent hypertrophic scars and keloids include silicone sheet and gel use, pressure and compression therapy, intralesional corticosteroid injections, laser therapy, radiotherapy, and cryotherapy 3-5. Various innovative pharmacologic modalities, such as calcium channel blockers, antiproliferative agents, interferons, retinoids, and immunotherapeutic agents, have also been introduced as topical or intralesional treatments 2, 6-8. Nevertheless, clinical outcomes remain inconsistent, and optimal regimens that yield satisfactory results have yet to be established.

The renin-angiotensin-aldosterone system (RAAS) plays an important role in the pathogenesis of organ fibrosis 9. Angiotensin II (Ang II), a core component of the RAAS, significantly influences blood pressure regulation and contributes to fibrogenesis in organs such as the heart and kidney 10. Skin tissue also possesses a complete RAAS, which plays a vital role in wound repair and tissue reconstruction through Ang II activity 11. Clinical and animal studies have reported the effects of topical or systemic administration of angiotensin-converting enzyme (ACE) inhibitors on pathological scars 12-16. Despite these findings, optimal delivery methods for ACE inhibitors in scar management remain unexplored. Silicone gel is a well-established treatment for scars, providing hydration, occlusion, and improved skin barrier function, while also serving as an effective transdermal delivery medium. Therefore, the combination of silicone gel and ACE inhibitors, such as captopril, is promising owing to their complementary mechanisms: silicone's ability to enhance drug penetration and modulate scar hydration and occlusion and captopril's targeted inhibition of Ang II-mediated fibrotic pathways. However, no studies have compared whether the topical application of silicone and ACE inhibitor gels produces enhanced effects. Our study addresses this gap by evaluating the combined effects of these treatments on pathological scar reduction.

We aimed to compare the scar-reducing effects of the topical application of silicone and captopril (an ACE inhibitor) gels, both individually and in combination. We demonstrated their enhanced efficacy in reducing scar formation in a rabbit ear hypertrophic scar model.

## Materials and Method

### Experimental setting and animals

Eight young adult female New Zealand White (NZW) rabbits, aged ≥ 5 months and weighing 2.5-3.5 kg, were used. The NZW rabbits were housed individually in cages under controlled environmental conditions (22-24 °C, 40-60% relative humidity) with a 12-h light/12-h dark cycle. Food and water were provided ad libitum.

### Experimental rabbit model

NZW rabbits were anesthetized with 35 mg/kg ketamine (Imalgine®, Merial, Lyon, France) and 5 mg/kg xylazine (Rompun®, Bayer, Leverkusen, Germany) and placed in a supine position. Five punch wounds were created on the ventral surface of each ear using an 8-mm biopsy punch. After complete excision of the epidermis, dermis, and perichondrium under a dissecting microscope, the bare cartilage was exposed. Hemostasis was achieved by manual compression, after which the wounds were covered with a polyurethane dressing (Tegaderm, 3M Health Care, St. Paul, MN, USA).

In total, 80 wounds were grossly examined at 1-day intervals for signs of infection, desiccation, and epithelialization and were photographed at 1-week intervals for evaluation. By day 17, all wounds had fully epithelialized. By day 28, hypertrophic scarring was successfully induced in all lesions.

### Preparation of the treatment agent

Captopril was used at a concentration of 50 mg/g, as pilot studies demonstrated phase separation at concentrations above this level. Lower concentrations (0.5 mg/g and 5 mg/g) were also evaluated during the pilot study as sequential 10-fold reductions from the maximum concentration. These evaluations confirmed that 50 mg/g was the optimal concentration, balancing stability and effectiveness.

Captopril gel (50 mg/g, 5% w/w) was prepared by mixing captopril powder (Sigma-Aldrich, St. Louis, MO, USA) with petrolatum (Sonneborn, LLC, Petrolia, PA, USA) and a vehicle composed of polyethylene glycol 400 (PEG 400; Fisher Scientific, Hampton, NH, USA), polyethylene glycol 4000 (PEG 4000; Sigma-Aldrich, St. Louis, MO, USA), and distilled water, added at 2-5%, 1-3%, and 5-10% w/w, respectively, to optimize viscosity and spreadability. The vehicle control consisted of the same base components with petrolatum, PEG 400, PEG 4000, and distilled water prepared without the active ingredient. The captopril-embedded silicone formulation was prepared by incorporating captopril (Sigma-Aldrich, St. Louis, MO, USA) at 50 mg/g into medical-grade silicone resin (Dow Corning®, Midland, MI, USA) along with the same base components. All formulations were homogenized to ensure uniform distribution and evaluated for stability and spreadability before use.

### Treatment

The wounds (n = 80) were randomly categorized into five groups (control, vehicle, silicone, captopril [CP50], and silicone + captopril [silicone + CP50]), with 16 wounds per group. From days 28 to 56, wounds were treated with vehicle, silicone, captopril 50 mg/g, or captopril-containing silicone gel, while untreated wounds served as controls.

### Tissue preparation

After 4 weeks of drug application (day 56), all rabbits were euthanized, and the scar tissues were harvested with a 0.5-cm margin of surrounding unwounded tissue. The specimens were bisected at the point of maximum hypertrophic scar height, as determined by gross examination. All specimens were fixed in 10% neutral buffered formalin, followed by dehydration, paraffin embedding, sectioning at 5-µm thickness, and hematoxylin and eosin staining for histopathological examination.

### Histopathological examinations

The tissue specimens were examined histopathologically, and the results were quantified based on the scar elevation index (SEI), fibroblast counts, capillary counts, and epidermal thickness index (ETI).

For histopathological examination, digital images were obtained using the Leica Application Suite Software LAS V 4.1 (Leica Microsystems, Wetzlar, Germany). The degree of scar hypertrophy was evaluated using the SEI, an indicator of cell proliferation, epithelial thickness, and matrix deposition. The SEI was calculated as the ratio of the total scar area to the area of normal tissue beneath the hypertrophic scar 17. The SEI was measured twice by a blinded examiner, and the two values were averaged. Fibroblast counts per three randomly chosen 1-mm2 fields were counted under 400× magnification. Capillary counts per field were quantified at 40× magnification by two histopathologists blinded to the group allocation. Epidermal hypertrophy degree was expressed as the ETI and was determined by measuring the thickness of the epidermis in the uninjured skin and epidermis of the scar at 400× magnification 18.

### Immunohistochemistry

Immunohistochemical analysis was performed on 4-μm-thick formalin-fixed, paraffin-embedded tissue sections using a Benchmark Ultra autostainer (Roche Tissue Diagnostics, Tucson, AZ, USA) and an UltraViewTM Universal DAB detection kit (Ventana, Oro Valley, AZ, USA), in accordance with the manufacturer's instructions. Paraffin sections were immunostained with primary antibodies against collagen type I (diluted 1:100, monoclonal 3G3; Abcam, Cambridge, UK), collagen type III (diluted 1:100, polyclonal ab7778; Abcam), Ki-67 (diluted 1:100, clone 30-9, Ventana), and vascular endothelial growth factor (VEGF; diluted 1:200, ab1316; Abcam). The scar tissue was evaluated using a semi-quantitative approach. For collagen types I and III, immunostaining was evaluated based on the proportion of positively stained cells as follows: (-) absent; (1+) weak; (2+) moderate; and (3+) strong staining. Final scores for each collagen type were calculated as the average staining intensity across three randomly selected fields at 100× magnification.

For Ki-67, 100 basal cells were counted in three randomly selected fields per tissue section to calculate the Ki-67 proliferation index 19 defined as the average percentage of Ki-67-positive nuclei in these fields.

VEGF expression was assessed semi-quantitatively by evaluating both staining intensity and the distribution of positively stained cells in five randomly selected scar fields per sample. Staining intensity was scored as 0 (negative), 1 (weak), 2 (moderate), or 3 (strong). The percentage distribution of positive cells was scored as 0 (< 10%), 1 (11-50%), or 2 (> 50%). The final VEGF expression score was calculated as the sum of the intensity and distribution scores (range, 0-5). A VEGF expression score of > 4 was classified as strong expression, whereas a score of ≤ 4 was classified as weak expression 20.

### Statistical analysis

Data were summarized using either medians with interquartile ranges or means with standard deviations, as appropriate. Differences among groups were analyzed using the Kruskal-Wallis test with Dunn's post-hoc Bonferroni correction. A P value <0.05 was considered statistically significant. Statistical analyses were performed using R software (version 4.3.1; R Foundation for Statistical Computing, Vienna, Austria).

## Results

### Treatment with 50 mg/g captopril-containing silicone inhibits thickening of scar tissue

**Figure 1:**
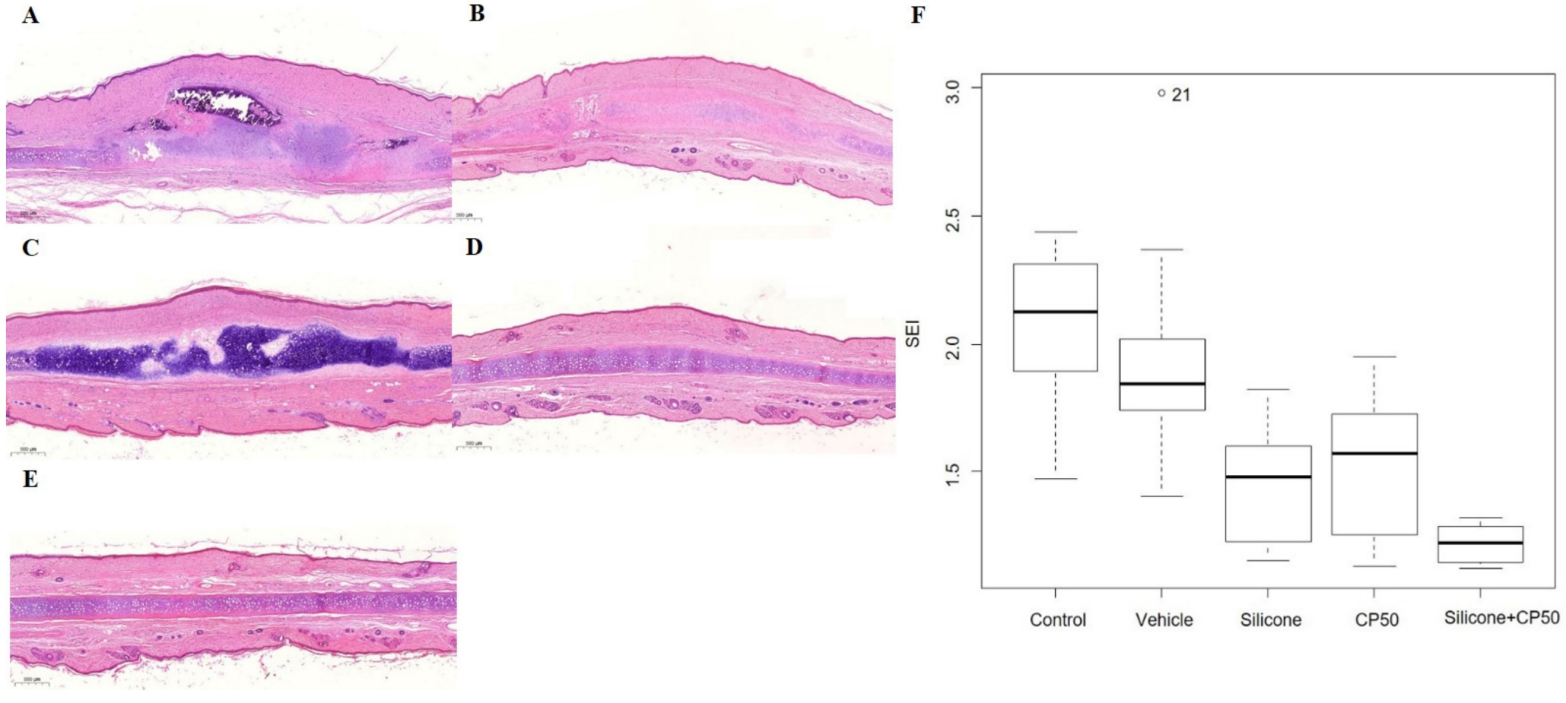
Histopathological findings (**A-E**, Hematoxylin & eosin, scale bar = 500 µm). **A** Control group showing fibroblast proliferation, with dense connective tissue, rich capillary lumina, and elevated scar. **B** The vehicle group shows similar results to the untreated control group. **C** The silicone group shows less prevalent fibroblast proliferation, a more diffuse distribution of loose connective tissue, and less scar elevation than the control and vehicle groups. **D** The CP50 group shows findings comparable to those of the silicone group. **E** The silicone + CP50 group shows no notable hypertrophic scar. **F** The median SEI differed significantly among the five groups (Kruskal-Wallis test; *P <* 0.0001). SEI, scar elevation index; CP50, captopril 50 mg/g; Silicone + CP50, 50 mg/g captopril-containing silicone.

Histopathological analysis revealed that hypertrophic scar areas in the untreated control and vehicle groups contained dense connective tissue, a less organized collagen structure, and abundant capillary lumens. Compared with the CP50 and silicone groups, the silicone + CP50 group exhibited flatter scars with reduced height, fewer fibroblasts, loosely arranged collagen bundles, and fewer capillary lumens. The median SEI differed significantly among the five groups (P < 0.0001). However, no significant differences were observed among the control, vehicle, silicone, and CP50 groups. Among all five groups, the SEI was significantly lower in the silicone + CP50 group than in the other groups (Figure 1, Table 1).

### Treatment with 50 mg/g captopril-containing silicone inhibits fibrosis

Significant differences in the median ETI and fibroblast count were observed among the five groups (P < 0.0001). However, no significant differences were found between the control and vehicle groups or between the silicone and CP50 groups. Among the five groups, ETI and fibroblast counts were significantly lower in the silicone + CP50 group than in the remaining groups.

**Figure 2:**
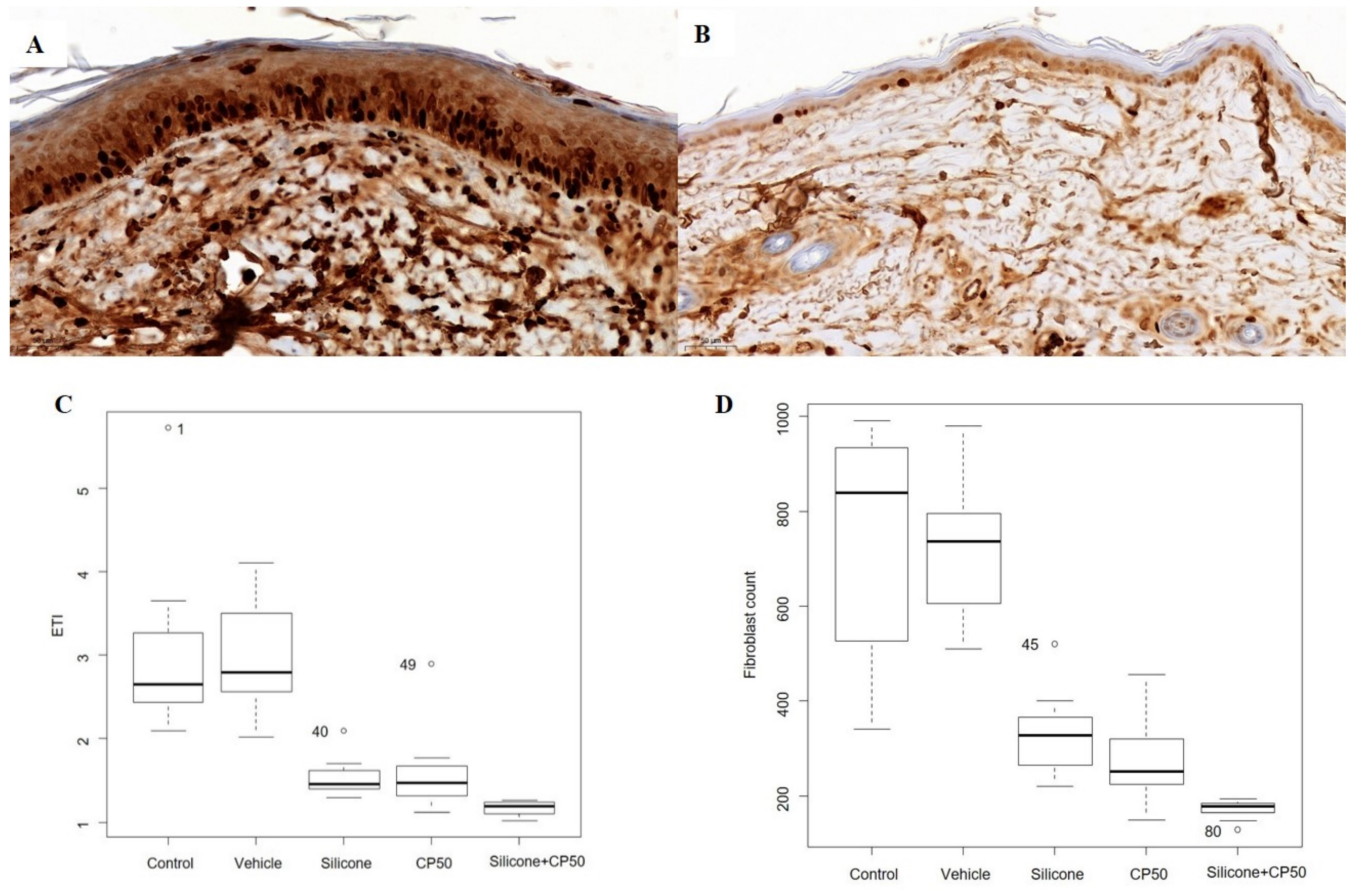
Immunostaining to assess the Ki-67 proliferation index (**A-B**, scale bar = 50 µm). **A** Strong Ki-67 immunoreactivity is seen in the tissues of the untreated control group. **B** Weak Ki-67 immunoreactivity is seen in the tissues of the silicone + CP50 group. **C** The median ETI in each group. **D** The median fibroblast count in each group. The median fibroblast counts and ETI differed significantly among the five groups (Kruskal-Wallis test; *P <* 0.0001). CP50, captopril 50 mg/g; Silicone + CP50, 50 mg/g captopril-containing silicone; ETI, epidermal thickness index.

The Ki-67 proliferation index also differed significantly among the five groups (P < 0.0001) and was significantly lower in the silicone, CP50, and silicone+CP50 groups than in the control and vehicle groups. However, no significant differences were observed among the silicone, CP50, and silicone+CP50 groups (Figure 2, Table 2).

### Treatment with 50 mg/g captopril-containing silicone inhibits scar deposition by inhibiting capillary count and VEGF expression

The median capillary count differed significantly among the five groups (P < 0.0001). However, no significant differences were observed between the control and vehicle groups or between the silicone and CP50 groups. Among all five groups, the median capillary count was significantly lower in the silicone + CP50 group than in the other groups.

**Figure 3:**
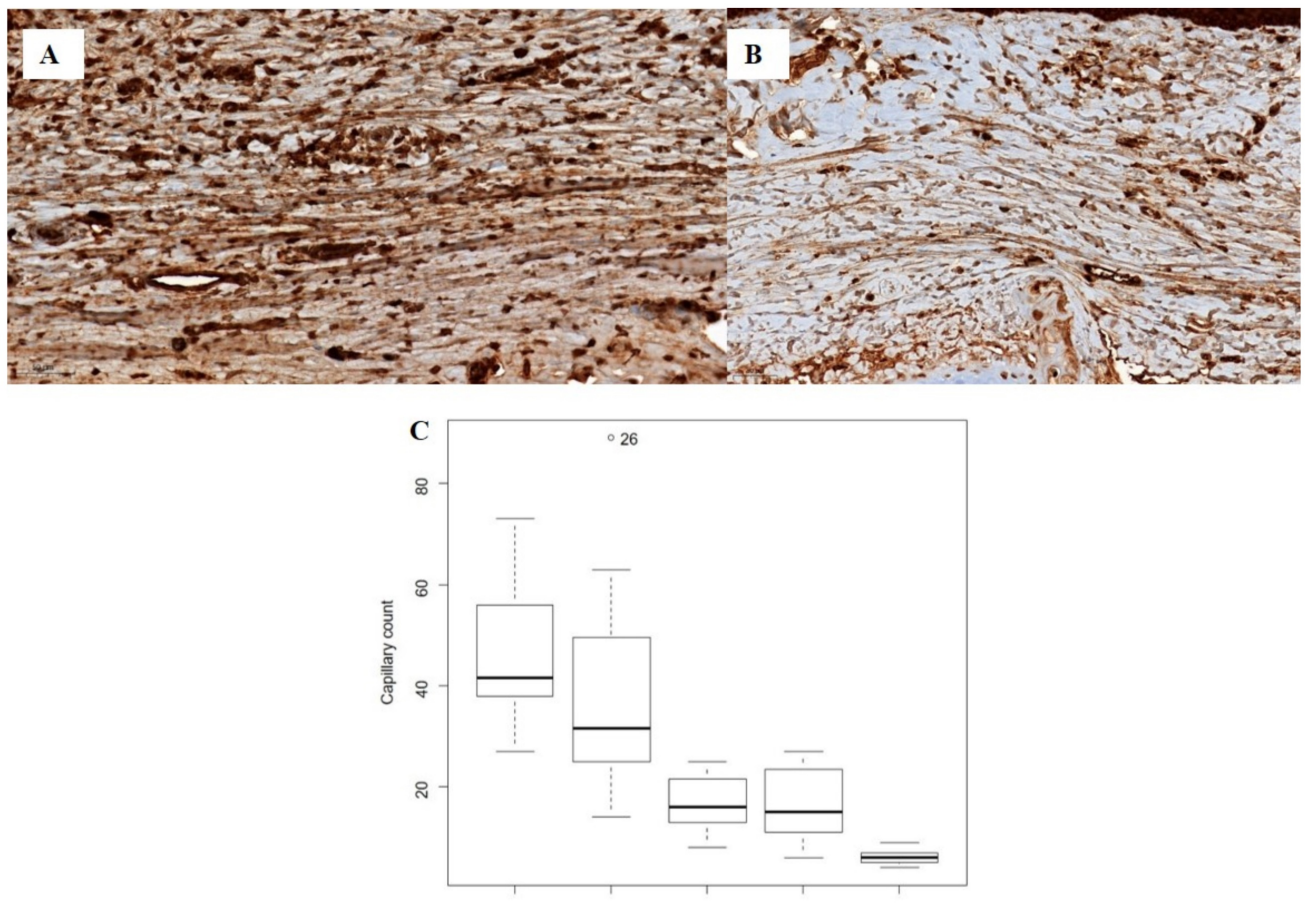
Immunostaining for VEGF (**A-B**, scale bar = 50 µm). **A** Strong VEGF immunoreactivity is observed in the tissues of the control group. **B** Weak VEGF immunoreactivity is observed in the tissues of the silicone + CP50 group. **C** The median capillary counts differed significantly among the five groups (Kruskal-Wallis test; *P <* 0.0001). VEGF, vascular endothelial growth factor; CP50, captopril 50 mg/g; Silicone+CP50, 50 mg/g captopril-containing silicone.

The VEGF expression score also differed significantly among the five groups (P < 0.0001), with lower scores in the silicone, CP50, and silicone + CP50 groups than in the control and vehicle groups. However, no significant differences were observed among the silicone, CP50, and silicone + CP50 groups in VEGF expression scores (Figure 3, Table 2).

### Treatment with 50 mg/g captopril-containing silicone inhibits collagen deposition by inhibiting collagen types I and III

**Figure 4:**
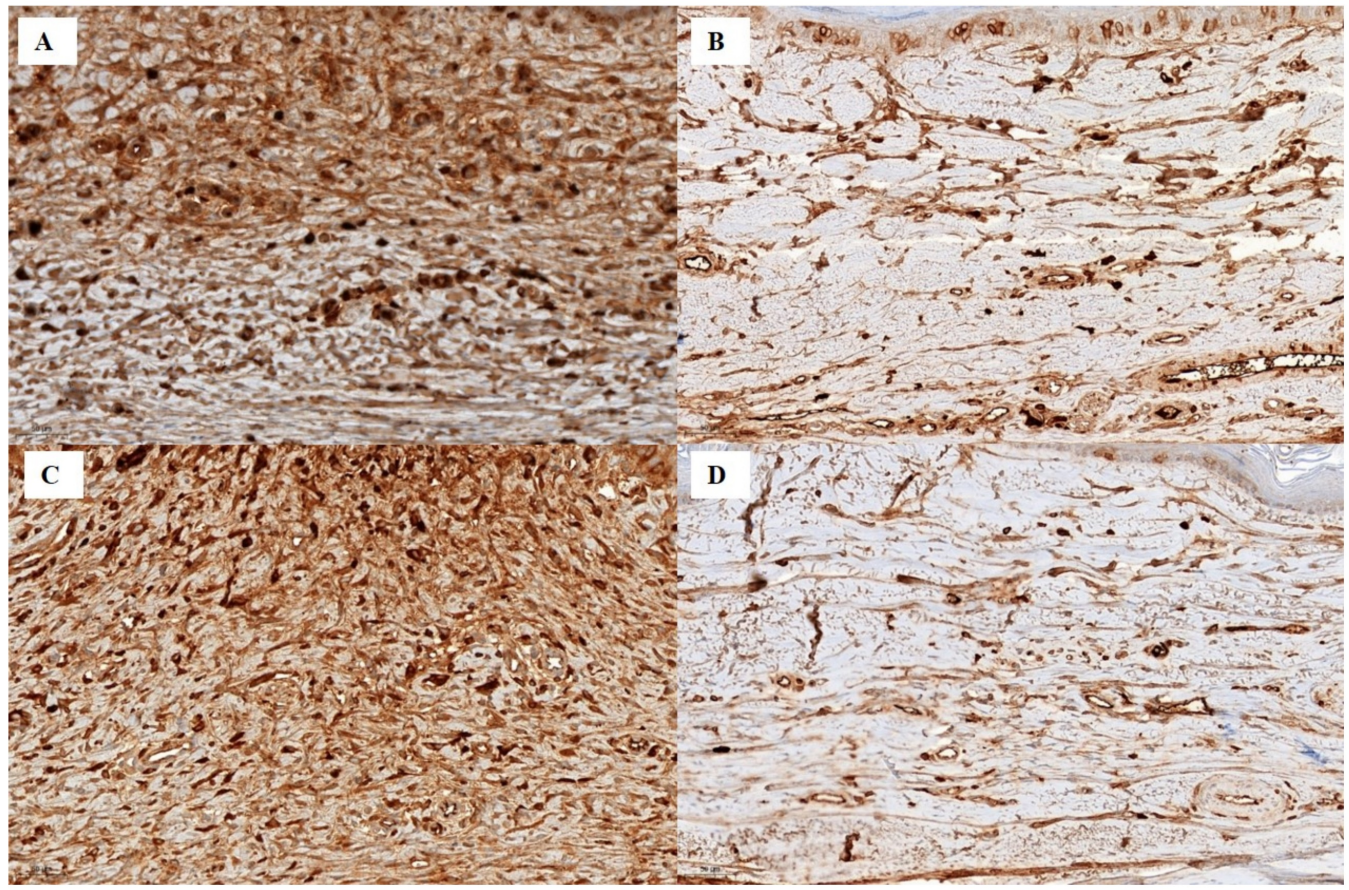
Immunostaining for collagen type I (**A-B**, scale bar = 50 µm). **A** Strong collagen type I staining is seen in the tissues of the control group. **B** Weak collagen type I staining is seen in the silicone + CP50 group. Immunostaining for collagen type III (**C-D**, scale bar = 50 µm). **C** Strong collagen type III staining is seen in the tissues of the control group. **D** Weak collagen type III staining is seen in the tissues of the silicone + CP50 group. Silicone + CP50, 50 mg/g captopril-containing silicone.

Collagen types I and III expression differed significantly among the five groups (P < 0.0001). Expression levels were significantly lower in the silicone and CP50 groups than in the control and vehicle groups. Among all five groups, collagen type I and III expression were significantly lower in the silicone + CP50 group than in the remaining groups. However, no significant differences were observed between the silicone and CP50 groups or between the control and vehicle groups (Figure 4, Table 2).

## Discussion

This study demonstrated the therapeutic effect of the topical application of the ACE inhibitor captopril, as well as the enhanced effect of captopril and silicone on hypertrophic scars in a rabbit ear model of hypertrophic scarring.

ACE converts Ang I into Ang II 21. Ang II plays a potent role in blood pressure regulation and fibrosis in some organs, including the heart and kidney, and is the core component of the RAAS 22. Recent research has also demonstrated the presence of a local RAAS in healthy skin. This cutaneous RAAS plays a vital role in wound repair and tissue reconstruction through Ang II 11, 23, which stimulates the proliferation, migration, and matrix production of human dermal fibroblasts 24, 25, as well as keloid pathogenesis via activation of the angiotensin type I receptor-dependent signaling pathway 26. Tan et al. 27 demonstrated that ACE-knockout mice and ramipril-treated wild-type mice exhibited narrower scar widths due to reduced fibroblast proliferation and decreased collagen deposition through the SMAD and transforming growth factor-beta-activated kinase one pathways. Systemic treatment with the ACE inhibitor enalapril reduced hypertrophic scar formation in a rabbit ear wound model by decreasing profibrotic collagen type III expression 14. Recent studies have also shown that the ACE inhibitor captopril effectively reduces scarring in genetically hypertensive rats compared with normotensive rats 13. In addition, topical treatment with ACE inhibitors has been shown to reduce scar hypertrophy and redness 28. Akershoek et al. 15 described the effects of both systemic and topical captopril administration during the early wound healing process on partial-thickness contact burns in a rat model.

Silicone gel reduces various scar characteristics, including elasticity, color, hardness, extensibility, height, smoothness, elevation, blood flow, volume, pruritus, redness, thickness, pliability, and pigmentation 29-31. Silicone gel exerts its effects primarily through hydration and occlusion. The occlusive effect of silicone leads to epidermal overhydration, altering the cellular morphology of the stratum corneum to a rounder shape, thereby facilitating the transfer of drug molecules into deeper layers 32. This mechanism reduces water loss from the scar, restores homeostasis to the scar, and reduces capillary hyperemia. The reduction in capillary activity further reduces collagen deposition by modulating keratinocyte activity, which in turn influences skin fibroblasts 30, 32. As the stratum corneum layer is considered a significant obstacle for transdermal drug delivery, enhancing transfer across this cellular barrier using silicone media would be effective for the transdermal delivery of captopril-containing silicone gel.

In this study, histopathological data showed that treatment with 50 mg/g captopril-containing silicone reduced scar tissue more effectively than silicone or 50 mg/g captopril alone. This finding highlights the enhanced effect of combining captopril 50 mg/g with silicone gel on scar tissue. Silicone and 50 mg/g captopril alone also effectively reduced scar tissue compared with the control and vehicle groups. However, no significant differences were found between these two groups. This result suggests that the reducing effect of the ACE inhibitor on pathological scarring was not inferior to that of the previous silicone gel. The 50 mg/g captopril gel reduced scar height and volume and inhibited fibroblast proliferation and collagen density. The hydration effect of captopril and silicone gel also contributed to the reduction of pathological scarring.

Collagen is secreted primarily by fibroblasts and is the most abundant protein in the extracellular matrix 33, 34. Compared with other collagen subtypes, collagen types I and III both increase hypertrophic scar formation, with a concomitant decrease in collagenase production 35, 36. In the present study, collagen type I and III expression in the treated groups (silicone, CP50, and silicone + CP50) was significantly lower than that in the control and vehicle groups. Furthermore, the silicone + CP50 group showed the lowest collagen type I and III expression among the treated groups. This result was consistent with the SEI and fibroblast counts observed in the histopathological analysis. The silicone + CP50 combination also reduced inflammatory cell infiltration and scar volume and improved scar pliability.

One of the key regulatory mechanisms controlling the synthesis of collagen types I and III is the transforming growth factor (TGF)-β/Smad signaling pathway 37. Aberrant activation of this pathway promotes excessive myofibroblast proliferation and abnormal accumulation of extracellular matrix components, including collagen types I and III, ultimately contributing to hypertrophic scar formation 38. In the present study, the silicone + CP50 combination demonstrated a significant reduction in collagen expression among the treatment groups. Given that Ang II signaling promotes TGF-β activation 39, the downregulation of Ang II by captopril likely contributed to reduced TGF-β expression, which in turn may have led to decreased collagen synthesis. This finding suggests that the combined therapy may exert antifibrotic effects, at least in part, by attenuating the TGF-β/Smad signaling pathway.

Ki-67 immunostaining was evaluated as a marker of epidermal proliferation 35. In this study, the ETI and Ki-67 immunostaining were significantly lower in all treated groups (CP50, silicone, and silicone + CP50) than in the control and vehicle groups. Among these, the silicone + CP50 group demonstrated the greatest reduction in the ETI. However, no significant differences in Ki-67 expression were observed among the treated groups. These findings support the concept that increased epidermal thickness in pathological scars is not necessarily associated with increased epidermal proliferation. Previous studies of mature keloid scars have shown that epidermal thickening does not result from hyperproliferation but may instead reflect abnormalities in early terminal differentiation affecting stratum corneum (SC) formation 19. This mechanism is characterized by aberrant expression of the early differentiation marker involucrin, which displays a panepidermal distribution and may extend prominently into the suprabasal layers or even the basal layer in keloid tissue 19, 40. Therefore, the superior reduction in the ETI observed in the silicone + CP50 combination group may be attributed to enhanced correction of this underlying abnormal terminal differentiation pathway. The combined therapeutic effect through silicone's established role in improving SC barrier function and captopril's potential paracrine modulation may help normalize the aberrant accumulation and disorganization of the SC, thereby reducing epidermal thickness independently of the proliferation rate.

Capillary overexpression was observed in the control and vehicle groups, whereas a significant reduction in capillary count was noted in the silicone + CP50 group. Gross examination revealed that this reduction was associated with decreased redness in the silicone + CP50 group. VEGF is a positive regulator of angiogenesis during wound healing, and VEGF overexpression has been associated with pathological scarring. Reduced VEGF levels have been observed in treated scar tissue 41. No significant difference in VEGF expression was found among the treated groups; however, the silicone + CP50 group showed the lowest value. The decrease in VEGF expression in the CP50-treated group may be associated with Ang II downregulation, as Ang II promotes neovascularization and the release of VEGF and platelet-derived growth factor 23, 42.

This study had certain limitations. First, the findings were based solely on histopathological analysis, with no supporting chemical studies such as Western blotting or polymerase chain reaction. These advanced molecular analyses could have provided deeper insights into the mechanisms of scar reduction; however, the robust histological results presented here strongly demonstrate the efficacy of the captopril-containing silicone gel. Further studies incorporating molecular assays are warranted to validate these findings. Second, this was an animal study using a rabbit ear hypertrophic scar model. While the rabbit ear model closely mimics hypertrophic scarring in humans compared with other rodent models, inherent limitations remain in directly translating these results to clinical practice. Additional research in humans is essential to confirm the clinical applicability of captopril for scar management. Finally, the long-term effects and potential systemic absorption of topical captopril were not assessed in this study, necessitating further investigation to ensure safety and sustained efficacy.

In conclusion, the topical application of captopril (50 mg/g) effectively reduced scar tissue in a rabbit ear model. Furthermore, the combination of captopril 50 mg/g and silicone exhibited the most significant effect in lowering hypertrophic scars by decreasing vascularity, pliability, and scar height. Therefore, captopril could serve as an alternative treatment option for scar management. Further studies are necessary to elucidate the mechanisms underlying the enhanced action of ACE inhibitors combined with silicone and transdermal drug delivery systems in pathological scars using established animal scar models and multiple evaluation parameters.

## Figures and Tables

**Table 1:** Summary of the histopathology data.

Group	1 (Control)	2 (Vehicle)	3 (Silicone)	4 (CP50)	5 (Silicone + CP50)	P-value	Post-hoc
	(N = 16)	(N = 16)	(N = 16)	(N = 16)	(N = 16)		
SEI	2.1 [1.9-2.3]	1.8 [1.8-2.0]	1.5 [1.2-1.6]	1.6 [1.3-1.7]	1.2 [1.1-1.3]	< 0.001	5<3=4<2=1
Fibroblast Count	838.5 [537.5-923.8]	735.0 [607.5-787.5]	327.5 [267.5-357.5]	250.0 [224.5-305.0]	177.5 [165.5-182.8]	< 0.001	5<3=4<2=1
Capillary Count	41.5 [38.0-56.0]	31.5 [25.0-44.3]	16.0 [13.0-21.3]	15.0 [11.5-22.8]	6.0 [5.0-7.0]	< 0.001	5<3=4<2=1
ETI	2.6 [2.5-3.3]	2.8 [2.6-3.5]	1.5 [1.4-1.6]	1.5 [1.3-1.7]	1.2 [1.1-1.2]	< 0.001	5<3=4<2=1

All data are expressed as medians and interquartile ranges. SEI, scar elevation index; ETI, epidermal thickness index; CP50, captopril 50 mg/g; Silicone + CP50, 50 mg/g captopril-containing silicone. Statistical significance at P < 0.05, based on the Kruskal-Wallis test, followed by a post-hoc Bonferroni correction for multiple comparisons.

**Table 2:** Summary of the immunohistochemical data.

Group	1 (Control)	2 (Vehicle)	3 (Silicone)	4 (CP 50)	5 (Silicone + CP 50)	*P*-value	Post-hoc
	(N = 16)	(N = 16)	(N = 16)	(N = 16)	(N = 16)		
Ki-67	2.44 ± 0.51	2.06 ± 0.68	1.56 ± 0.63	1.25 ± 0.58	1.31 ± 0.48	< 0.001	5=4=3<2=1
Collagen Type I	2.69 ± 0.48	2.38 ± 0.72	1.88 ± 0.34	1.69 ± 0.70	1.38 ± 0.50	< 0.001	5<3=4<2=1
Collagen Type III	2.69 ± 0.48	2.44 ± 0.51	1.75 ± 0.45	1.75 ± 0.68	1.25 ± 0.45	< 0.001	5<3=4<2=1
VEGF	2.81 ± 0.40	2.63 ± 0.50	1.69 ± 0.48	1.56 ± 0.63	1.25 ± 0.45	< 0.001	5=4=3<2=1

All data are expressed as means and standard deviations. VEGF, vascular endothelial growth factor; CP50, captopril 50 mg/g; Silicone + CP50, 50 mg/g captopril-containing silicone. Statistical significance at *P* < 0.05, based on the Kruskal-Wallis test, followed by a post-hoc Bonferroni correction for multiple comparisons.

## Data Availability

The data supporting the findings of this study are available from the corresponding author upon reasonable request.

## Abbreviations

ACE: angiotensin-converting enzyme
Ang II: angiotensin II
ETI: epidermal thickness index
NZW: New Zealand White
RAAS: renin-angiotensin-aldosterone system
SEI: scar elevation index
VEGF: vascular endothelial growth factor
